# Novel technologies for the diagnosis of urinary tract infections

**DOI:** 10.1128/jcm.00306-24

**Published:** 2025-01-06

**Authors:** Tomas Bermudez, Jonathan E. Schmitz, Malcolm Boswell, Romney Humphries

**Affiliations:** 1 Department of Pathology, Microbiology, and Immunology, Vanderbilt University Medical Center 204907 , Nashville, Tennessee, USA; 2 Independent MicroDx Consultancy Services, Tuson, Arizona, USA; Boston Children's Hospital 1862 , Boston, Massachusetts, USA

**Keywords:** urinary tract infection, rapid diagnostics, urine culture, urinalysis

## Abstract

Urinary tract infections (UTIs) impose a substantial burden on patient quality of life and urine testing accounts for the majority of workload in many clinical microbiology laboratories. Traditional UTI diagnosis relies on symptoms, urinalysis, and culture which are interpreted based on historical guidelines. This approach, while foundational, presents limitations, particularly in complex cases. Low-level bacteriuria and the presence of fastidious organisms are often overlooked or entirely missed in standard urine culture, stressing the need for novel diagnostic methods and technologies. This mini-review summarizes the existing state of UTI diagnostics in 2024 and covers current and upcoming technologies including rapid molecular-based pathogen identification, next-generation sequencing, and advanced antimicrobial susceptibility testing. However, these methods represent unique challenges, and as they are implemented, they will require the field to adapt to new concepts to avoid misdiagnosis and overtreatment.

## THE CLINICAL LANDSCAPE OF URINARY TRACT INFECTIONS

An estimated 400 million urinary tract infections (UTIs) occur each year globally, with over 200,000 associated deaths, making UTIs one of the most common bacterial diseases and drivers of antimicrobial usage (1). UTIs disproportionately affect women, up to 60% of whom experience at least one UTI in their lifetime (2). This association is generally attributed to anatomy, although hormonal factors also play an increasingly recognized role, including in relation to the vaginal microbiome (3). Traditionally, a UTI is defined by clinical signs and symptoms such as urinary frequency, urgency, pelvic or lower abdominal pain, pyuria, or urinary incontinence—often in combination with identification of an offending pathogen via urine culture (4). This symptom-based approach to UTIs can lack sensitivity or specificity in certain populations, including the very young and elderly, or individuals who are chronically catheterized, with urologic or neurologic abnormalities. For these patients, signs and symptoms can be ambiguous (5).

UTIs are characterized according to several schema and terminologies, which are relevant to their diagnostic work-up (6, 7). Anatomically, *lower UTIs* are confined to the bladder (cystitis) and/or urethra, while *upper UTIs* involve ascension to the kidneys (pyelonephritis). The latter are more commonly associated with both bacteremia (a condition defined microbiologically) and urosepsis (defined clinicopathologically), although they may occur with cystitis alone (8). UTIs may be further defined according to patient risk factors. *Uncomplicated UTI* (uUTI) affects non-pregnant, immunocompetent females without structural/functional urinary tract abnormalities or other predisposing conditions. Notably, an explicit microbiologic diagnosis (i.e., urine culture) is not necessarily indicated for routine cases of uncomplicated cystitis, as empiric antimicrobial therapy is often appropriate (4). *Complicated UTI* (cUTI) is an umbrella term for infections arising in the context of risk factor(s) listed above for uUTI. Traditionally, a UTI in a male is inherently considered complicated (although this is controversial [9]). Complicated infections secondary to catheterization are termed *catheter associated UTIs* (10). In terms of temporal dynamics, multiple episodes of symptomatic infection (two within 6 months or three within 1 year) with intervening periods of clinical resolution are *recurrent UTIs* (11). These may represent persistent infections by the same offending organism or reinfection by a different species/strain. Likewise, the term *chronic UTI* is applied loosely to either recurrent or unresolving infections. Finally, the concept of *asymptomatic bacteriuria* (ASB) refers to recovery of a potential uropathogen at high burden (typically >10^5^ CFU/mL) in the absence of symptomatology (12). Antimicrobial therapy is typically not indicated for ASB, except in certain populations at high risk for adverse outcomes (e.g., renal transplant recipients and pregnant women, especially those undergoing invasive urologic procedures) (13).

UTIs are opportunistic infections, whereby pathogens ascend from colonizing niches (the gut, perineum, and/or vagina) into the urinary tract. *Escherichia coli* is the most common agent of UTIs, causing 65%–75% of infections overall. Beyond *E. coli*, the taxonomic landscape of UTIs is incredibly diverse (14–16). For uncomplicated infections, *Staphylococcus saprophyticus* and *Klebsiella pneumoniae* are also commonly observed. In the setting of cUTIs, when host comorbidities present added opportunities for infection, a broad array of bacteria are observed including *Pseudomonas aeruginosa*, other Enterobacterales (*Proteus*, *Serratia*, and *Enterobacter* spp.), and *Enterococcus* spp. For some microorganisms, the diagnostic significance in urine is not always clear and may be context dependent, including *Staphylococcus aureus* (17) and *Candida* spp. (18). The diagnostic *status quo* is likewise complicated by several rare, but well-established, uropathogens with more fastidious growth characteristics, and a growing list of species whose status as commensal-versus-pathogen is not always clear. The diagnostic challenge posed by such emerging uropathogens is detailed further below.

Against this complex background, traditional diagnostic cultures—together with urinalysis—represent staples of care (19). At the same time, there is a growing desire for UTI diagnostics that leverage new technology for more sensitive, specific, or rapid results. Of particular interest are tools that reduce the latency between initial, empiric UTI management and targeted therapy, based on species identification (ID) and antimicrobial susceptibility testing (AST). Various new platforms for UTI diagnosis are under development. The ultimate clinical impact of these tools will be a function of the method, interpretation, cost, regulatory status, population tested, and even the rapidly growing knowledge on urinary tract microbiology. To these ends, we provide a technical overview of these emerging technologies.

## URINE CULTURE AS A STANDARD OF CARE

Bacterial cultures have been the cornerstone to the diagnosis of UTIs since the 1950s. It is therefore important to understand the basis of these culture-based techniques, against which any new technologies are compared. Even before a urine specimen arrives in the lab, culture protocols are defined by pre-analytic steps that begin with collection. Into the early 1950s, urine specimens were more frequently collected via catheterization to reduce contamination, although the practice as conducted at the time not infrequently led to iatrogenic UTIs (20). Voided specimens have since become the norm, with a midstream clean catch required to minimize periurethral contamination. While first-morning specimens yield higher bacterial concentrations than samples collected later in the day, the impact on collection practices is likely minimal. If not processed within 30 minutes (21) to 2 hours (22), urine must be kept refrigerated at 4°C to minimize changes in bacterial composition. Boric acid (20 g/L) is commonly used as a bacteriostatic agent to stabilize specimens for up to 48 hours (23, 24).

Semi-quantitative urine culture is performed by most laboratories (25). For voided specimens, 1 µL of urine is plated onto a non-selective and Gram-negative-selective media which are incubated aerobically for a minimum of 12–18 hours, leading to growth of non-fastidious aerobes and facultative anaerobes. Over the years, various additional selective/differential media have been developed to aid in presumptive identification. Catheterized, cystoscopic, and aspirated suprapubic collections may be inoculated with 10 µL of specimen and plates held for up to 48 hours (22). The inoculation volume defines the lower limit of detection: 1,000 CFU/mL for 1 µL, 100 CFU/mL for 10 µL. Furthermore, the 1 µL–10 µL inoculum helps reduce potential co-isolation of periurethral contaminants, which rarely exceed 1,000 CFU/mL under proper clean-catch conditions (26). Plates are observed for the quantity and purity of microbial growth, which are both summarized in the reported result. Higher-abundance growth is more strongly associated with clinical significance, while multiple colony morphotypes (typically ≥3) are considered evidence of periurethral contaminant. At the same time, the latter phenomenon must be weighed alongside the possibility of polymicrobial UTIs in at-risk patients (27).

Along with abundance and purity, additional factors also dictate how each colonial morphotype is characterized by the laboratory. These include the method of collection (invasive versus non-invasive), characteristics of the patient (pediatric versus adult, etc.), as well as the identity of the organisms themselves. Species generally considered urogenital flora (e.g., viridans *Streptococcus* and coagulase-negative staphylococci other than *S. saprophyticus*) are infrequently pursued. Various guidelines synthesize these parameters as a framework for working up isolates, including whether to perform definitive taxonomic ID and phenotypic AST (25). Typically, complete ID/AST is performed for each of one or two cultured urinary isolates. For voided urine, isolation of a uropathogen at >10^5^ CFU/mL is interpreted as the highest evidence of clinical significance, a threshold that shifts down by 1 log for invasively collected specimens. With properly collected specimens, diagnostic cultures from healthy individuals typically generate no colonies (cases of ASB notwithstanding), yielding a reference value of *No Growth Observed*.

Although guidelines help ensure intra-laboratory standardization, traditional culture protocols are beset by key shortcomings. First and foremost, one must recognize the workflow limitations of culture in relation to diagnostic turnaround and clinical impact. Initial culture data are not available the same day as specimen collection, and traditional phenotypic AST adds at least 1 more day to final reporting. By the time definitive laboratory results can guide care, a patient may have already received several days of empiric therapy. Timeframe aside, the practice of inferring clinical significance from cultured bacterial concentrations stems from historical studies with circumscribed scope. A threshold of 10^5^ CFU/mL derives from work focusing on the reproducibility of semi-quantitative data in the context of ASB, as well as the progression of patients to pyelonephritis (11). Nevertheless, this same approach is now applied to virtually every patient population for whom a culture is desired (28). A urine culture is now even employed for motivations outside the diagnosis of UTI proper, including evaluation of patients for bladder pain syndrome/interstitial cystitis (29). As many laboratories attest, the broad application of a method that was initially developed around a narrow focus can led to poor sensitivity/specificity in many scenarios (5).

From a microbial perspective, each uropathogenic species may carry its own infection dynamics, such that employing universal thresholds across all species to define infection (e.g., ≥10^5^ CFU/mL) is likely an oversimplification (30, 31). Some patients may experience symptomatic infections with bacterial counts as low as 10^2^ CFU/mL (32). Not only are these levels below the limit of detection of a standard 1 µL inoculum, but they overlap with potential levels of contamination. Unfortunately, in deciphering low-level pathogens from prominent contaminants, a laboratory cannot directly control for the variable quality of specimen collection, and a traditional culture cannot differentiate the active pathological process of a UTI from colonization/ASB.

## URINARY DIAGNOSTICS IN THE MICROBIOME AGE

More fundamentally, interpretations of diagnostic urine culture are predicated on the laboratory conditions under which they are performed, that is, aerobically and with brief incubation times. In fact, these protocols long created the notion that urine is physiologically sterile, as a reference result of *No Growth Observed* assumes that bacteria are absent from a healthy bladder. Recent recognition of the human urobiome, even outside the context of ASB, has turned this assumption around (33–36). By applying extended culture conditions to urine—including larger inocula, microaerobic/anerobic/CO_2_-enhanced atmospheres, additional media, and longer incubation times—urine cultures frequently reveal more fastidious commensals that otherwise go unrecognized diagnostically (37). These same microbial populations can be observed/assessed by molecular metagenomics, most notably 16S-based techniques (38). Enhanced techniques are better suited to identify certain uncommon, or potentially underrecognized, bacterial agents of UTI. With variable supporting evidence—from individual case reports to larger bodies of literature—these atypical uropathogens include *Aerococcus urinae*, *Actinotignum schaalii*, *Alloscardovia omnicolens (39)*, lipophilic *Corynebacterium* spp. (in particular, *Corynebacterium urealyticum*) (40), *Gardnerella vaginalis* (41), *Bordetella hinzii* (42)*,* among others.

Challenges do arise, however, around the possibility of applying metagenomic techniques to routine practice. In addition to potential uropathogens, these approaches identify a diversity of nonpathogenic commensals, greatly complicating reporting and interpretation on a case-by-case basis. From the perspective of the urobiome, low-level ASB may be viewed as a physiologic norm instead of an outlying phenomenon, and *No Growth Observed* should no longer be considered a universal reference value. Complicating matters further, many of the species now viewed as emerging uropathogens can be detected in both subjects with and without UTI symptoms (43). Important avenues of research have emerged around the human urobiome, including how its population structure differs between individuals, evolves over time, and impacts other urologic conditions. In this light, the presence/abundance of certain species may be variably associated with clinical symptomatology, as modified by host factors and other commensals. This “pathobiome” view of the urinary tract is far more nuanced than the traditional binary relationship between organism and disease (i.e., *presence = infection, absence = health*). While standardized methods for urobiome characterization have been proposed (44), these techniques have not supplanted traditional cultures within most clinical laboratories. As with other areas of the microbiome, outstanding questions remain on how urobiome data can be leveraged in a manner that is both evidence-based, as well as in the context of personalized care (45).

## NOVEL TECHNOLOGIES IN THE DIAGNOSTIC PIPELINE

Given the challenges associated with traditional methods, it is perhaps unsurprising that the diagnostic field is pursuing solutions through novel technology. Market analysis demonstrates that over 30 direct-from-specimen products are in development for rapid detection, ID, phenotypic AST, and/or resistance gene detection for urinary microbes. The methods can be categorized into methods that screen for bacteriuria, multiplex nucleic acid amplification tests, next-generation sequencing (NGS) tests, and novel phenotypic AST approaches, summarized in Table 1. Selected tests that are in later development are described herein, and in Tables 2 to 4. In addition to these, many reference laboratories have started to offer testing based on next-generation techniques broadly, as outlined in Table 5.

**TABLE 1:** Summary of novel UTI diagnostic technologies

Method	Detection principle	Pros	Cons	References
Bacteriuria screen assays	Detects surrogate markers of infection; non-specific bacterial detection through light scattering technology	• High negative predictive value for positive urine culture	• Low positive predictive value of infection	(46–54)
		• Can be used to enhance direct- from-urine MALDI-TOF identification	• Does not reveal pathogen ID	
Rapid molecular identification	Targets pathogen DNA/RNA; multiplex PCR options	• Rapid results	• Panels may have limited targets	(55–58)
		• Antimicrobial resistance gene targets are included in most assays	• Variable LOD between assays	
		• Better able to detect fastidious organisms	• Will not differentiate between pathogen and urinary microbiota	
Next-generation sequencing	Detects all bacteria present via sequencing	• Most beneficial for patients of complicated or recurrent UTI	• High likelihood of false-positive results	(59–63)
		• Can detect non-culturable microorganisms	• Not ideal for patients with uncomplicated UTI	
		• Antimicrobial resistance gene targets are included in most assays	• Majority are offered only by reference laboratory models	
Rapid phenotypic susceptibility tests	Measures growth in the presence of antibiotics	• Rapid evaluation of antimicrobial susceptibility patterns	• Does not reveal pathogen ID; current CAP accreditation standards require AST to be linked to a specific organism	(64–70)
		• Innovation in methodology: single-cell imaging, light scattering, detection of enzymatic activity	• Variability in scope of susceptibility targets between assays	

**TABLE 2:** Summary of novel urine screening assays for prediction of bacteriuria^*a*^

Test	Manufacturer	Regulatory status	Technology	AST method	Time to results	Specimen	Throughput	LOD	Platform	Point of care design?
216Dx UTI System	BacterioScan	IVD	Laser light scattering	Possible	3 h	Urine	20 specimens/day	50,000 CFU/mL	216Dx UTI System	No
Rapid Urine Culture Test	Alifax	CE-IVD	Light scattering	Possible	<4 h	Urine	60–420 specimens	30,000 CFU/mL	Alfred 60/AST or HB&L UROQUATTRO	No
Uriscreen	Savyon Diagnostics Ltd.	CE-IVD	Detects catalase activity as indicator of bacteria in somatic cells	N/A	2 min	Urine	One specimen	N/A	N/A	Yes
UTI-lizer Digital Dipstick	Utilizer	RUO	Miniaturized culturing in a digital bioassay	N/A	8 h	Urine	One specimen	253 CFU/mL	N/A	Yes
UTRIPlex	Global Access Diagnostics	RUO	Matrix metalloproteinase-8 and human neutrophil elastase detection via lateral flow	N/A	6 min	Urine	One specimen	N/A	N/A	Yes
Urine screen	Pattern Biosciences	RUO	Single-cell compartmentalization and imaging	Possible	2 h	Urine	One specimen	N/A	Digital Culture Platform	No

^*a*^ AST, antimicrobial susceptibility testing; LOD, limit of detection; CFU, colony forming unit; CE-IVD, Conformité Européenne *in vitro* diagnostic; IVD, US Food and Drug Administration cleared *in vitro* diagnostic device; RUO, research use only; N/A, not applicable.

**TABLE 3:** Selected nucleic acid-based tests in development for urine specimens^*a*^

Test	Manufacturer	Regulatory status	ID method	AMR gene(s) detected	Bacterial ID target(s)	Fungal ID target(s)	Parasites	Viruses	Time to results	Specimen	Throughput	LOD	Platform	Point of care design?
**DNA/RNA hybridization**														
CAPTURE UTI	GeneCapture	RUO	Unamplified RNA Expression “CAPTURE” Technology	0	6	1 *Candida* spp.	0	0	2 h	Urine	40 specimens per run	10,000 CFU/mL	GeneCapture	No
**Nucleic acid amplification tests**														
CLoNeT *E. coli* Assay for UTI	ID Genomics	RUO	Multiplex PCR with lateral flow detection	0	*E. coli*	0	0	0	<45 min	Urine	One specimen	100–1,000 CFU/mL	Multiple RT PCR thermocyclers	Yes
Lodestar UTI Test	Llusern Scientific	UK CA	LAMP	0	6	0	0	0	0.5 h	Urine	One specimen	10,000 CFU/mL	Llusern System - Lodestar DX analyzer	Yes
Multiple Primer Sets Targeting UTI Pathogens	BioGX	RUO	Multiplex RT-PCR	11	42	8	1	0	2 h	Urine (neat or boric acid preserved)	24–96 specimens	ND	BD Max, ABI, or Bio-Rad	No
OpenArray Urinary Tract Microbiota	ThermoFisher	RUO	Multiplex PCR	0	16	1 *Candida* spp.	0	0	5 h	Urinary tract samples	48–192 specimens	ND	QuantStudio 12K Flex Instrument with OpenArray block (OpenArray AccuFill System)	No
PathoKey MP UTI ID & AMR PCR Test	Vela Diagnostics	RUO	Multiplex RT-PCR	14	13	1 *Candida* spp.	0	0	≥4 h	Urine	40 samples (plus controls) per run	1000 copies/mL	Multiple RT PCR thermocyclers	No
QIAstat-Dx cUTI Plus AMR Panel	Qiagen	RUO	Multiplex RT-PCR	ND	ND	ND	ND	ND	1h	Urine	160 specimens/day	ND	QIAstat-Dx	No
Unyvero UTI Panel	Curetis GmbH	RUO	Multiplex PCR	15	23	4 *Candida* spp.	0	0	5 h	Urine (midstream, suprapubic, or fresh catheter specimens)	2–12 specimens	4,000–100,000 CFU/mL	Unyvero System	No
Usense	Module Innovations	RUO	Size-dependent signal amplification by nanoparticles	0	ND	0	0	0	15 m	Urine	One specimen	ND	ND	Yes
UTI Panel	Randox	RUO	Multiplex RT-PCR	8	23	1	0	0	<4 h	Urine	One specimen	10,000 CFU/mL	Vivalytic platform (point of care)	Yes
**Next-generation sequencing based assays**														
Urinary Pathogen ID/AMR Enrichment Kit	Illumina	RUO	Targeted DNA sequencing with target enrichment	3,728	121	14	4	35	1 day	Urine	384 specimens	Demonstrated to 100,000 CFU/mL	Illumina NGS instruments	No

^*a*^ ID, identification; AMR, antimicrobial resistance; LOD, limit of detection; RUO, research use only; UK CA, United Kingdom 851 Conformity Assessment; PCR, polymerase chain reaction; RT, real-time; LAMP, loop mediated amplification; ND, no data.

**TABLE 4:** Selected phenotypic antimicrobial susceptibility tests in development for rapid determination of antimicrobial susceptibility directly from urine specimens^*a*^

Test	Manufacturer	Regulatory status	ID included?	AST method	Antimicrobials tested	Time to results	Specimen	Throughput	Platform	Point of care design?
Astrego AST	Sysmex/Astrego	CE-IVD	No, general bacteriuria call	Single-cell imaging with nanofluidics	Amoxicillin-clavulanic acid; ciprofloxacin, fosfomycin, nitrofurantoin, trimethoprim	<30 min	Urine	One specimen	Astrego PA-100 AST System	Yes
ASTSense	Module Innovations	RUO	No, general bacteriuria call	Size-dependent signal amplification by nanoparticles	Not listed, many classes defined in patent application	2 h	Urine	One specimen	ASTsense system	Yes
iFAST	iFAST Diagnostics	RUO	No	Single-cell microfluidic impedance cytometry	Ampicillin, amoxicillin-clavulanate, cefoxitin, cefotaxime, ceftazidime, ertapenem, meropenem, imipenem, aztreonam, piperacillin-tazobactam, gentamicin, amikacin, ciprofloxacin, ceftolozane-tazobactam, ceftazidime-avibactam, fosfomycin, SXT	3 h	Urine, blood culture broth	25 specimens/day	iFAST System	No
JIDDU Test	Astek	RUO	No, general bacteriuria call	Detection of metabolic activity in presence of antimicrobials	SXT, ceftriaxone, levofloxacin, nitrofurantoin, cephalexin	<1 h	Urine	One specimen	JIDDU System	Yes
Rapid Urine Culture Test	Alifax	CE-IVD	No, general bacteriuria call	Light scattering growth curve evaluation	Not listed	<4 h	Urine	60–420 specimens	Alfred 60/AST or HB&L UROQUATTRO	No
U-DETECT	BioAmp Diagnostics	RUO	No	DETECT: Dual-Enzyme Trigger-Enabled Cascade Technology	Specific for detection of ESBL	<0.5 h	Urine	One specimen	None	Yes

^*a*^ AST, antimicrobial susceptibility testing; CE-IVD, Conformité Européenne *in vitro* diagnostic; ID, identification; RUO, research use only; SXT, trimethoprim/sulfamethoxazole.

**TABLE 5:** Selected reference laboratories offering next-generation urine diagnostic testing for microbial pathogens^*a*^

Reference laboratory test	Manufacturer	Regulatory status	ID method	AST method	AMR gene(s) detected	Bacterial ID target(s)	Fungal ID target(s)	Parasites	Viruses	Time to results	Specimen	LOD
Advantage Urinary Tract	Advanta	LDT	PCR	Possible with reflex to culture and AST	17	22	4	0	0	≤24 h	Urine	ND
Guidance UTI	Pathnostics	LDT	PCR	Pooled AST	32	26	4	0	0	<1 d	Urine	ND
Uroswab	Medical Diagnostics Laboratories	LDT	PCR	Not done	Unspecified “resistance gene profiling”	7	0	0	0	<3 d	Urine	ND
Urinary Tract Infection Panel	Core BioLabs	LDT	PCR	Not done	Eight markers, undefined	18	5	0	0	ND	Urine	ND
UroKEY	MicroGenDx	LDT	qPCR + NGS	Not done	Unspecified	>57,000	1	0	0	<3.5 d ID	Urine	ND
UTI Panel Molecular	Accu Reference Medical Lab	LDT	RT-PCR	N/A	27	15	1	0	0	1 business day	Urine (boric acid preserved)	ND
UTI Test	Clarity Lab Solutions	LDT	PCR	Pooled AST for 19 antimicrobials	3	19	5	0	0	<28 h	Urine	ND

^*a*^ ID, identification; AST, antimicrobial susceptibility testing; AMR, antimicrobial resistance; LOD, limit of detection; LDT, laboratory developed test; N/A not applicable, ND, no data.

### Bacteriuria screening assays

Urinalysis (UA) is traditionally used to aid with the diagnosis of UTI. This method screens for the presence of leukocytes and bacteria via the presence of nitrites and/or microscopic evidence of bacteria. The UA has a high negative predictive value for a positive culture (71), but suffers from low positive predictive value for infection. As such, improved bacteriuria screens can provide benefit by screening out the roughly one-third of urine specimens submitted to the clinical microbiology laboratory that are negative for bacterial growth. Such tests provide early directional information to the treating clinician, while reducing laboratory labor and costs. Next-generation screening methods include lateral flow immunoassays, flow cytometry, or light scattering technologies to achieve these same endpoints, but with potentially lower limits of detection than traditional UA (Table 2).

The sole novel Food and Drug Administration (FDA)-cleared method for rapid detection of bacteriuria is the 216Dx (BacterioScan, St. Louis, MO), which evaluates for the presence and quantity of bacteria in urine specimens using a proprietary light scattering method in 3 hours. Studies demonstrate a sensitivity of 96.5% and specificity of ~72% when compared to urine culture (46, 47). Outside the USA, the Uro-Quick (Alifax, Polverara, Italy) has been available for over a decade that also detects bacteriuria within 3 hours (Table 2) by applying light scattering technology (48). Both the Bacterioscan and Alifax technologies have been used to qualify specimens for a direct-from-urine mass-assisted laser desorption ionization time-of-flight mass spectrometry (MALDI-TOF MS) identification, by rapidly screening for specimens with sufficient cell mass for peak generation using standard MALDI-TOF MS in clinical laboratories (46, 49). The Jimini (Usense, Boulogne-Billancour, France) is a label-free device platform that applies gold-based electrodes immobilized with capture probes for both host inflammatory (interleukin-8, prostaglandin E2) and bacterial (lipopolysaccharide) targets within 5 minutes (50). This test is designed for point-of-care use and is being evaluated for AST as described further below.

The Uriscreen (Savyon Diagnostics, Ashdod, Israel) is a method developed that evaluates for the presence of catalase activity as a measure of bacteriuria. The test was evaluated to screen for ASB in pregnancy, but was found to have low sensitivity (60.7%) and specificity (89.3%) compared to culture, in a small study of 28 women (51). This test was also evaluated in a study of 300 pediatric patients in an ambulatory setting, which found a sensitivity of 67% and specificity of 69%, as compared to culture (52). The UTRIPlex (Global Access Diagnostics, Bedford, UK), which measures three inflammatory markers in urine, was evaluated in this same pediatric study and was associated with a sensitivity of 21% and specificity of 94% (52). The UTI-lizer (UTIlizer AB, Sweden) is a digital dipstick method that provides the opportunity for point-of-care culturing, whereby urine is applied to a series of isolated microreactors and incubated for 8 hours. Bacterial growth leads to a colorimetric change, based on the species, which is read visually using a principle akin to use of chromogenic media applied in clinical microbiology laboratories. The number of wells with color change can be used to quantify bacterial load in the urine specimen. Limited data suggest this method is associated with high negative predictive values (>97%) but positive predictive value versus culture ranged from 0 to 90%, based on microorganism present in the urine (53).

Finally, the single-cell technology in development by Pattern Biosciences (Austin, TX) is being evaluated for testing of urine specimens. This technology enables single-cell detection and quantification, through compartmentalization of bacterial cells in droplets and imaging analysis via fluorescence microscopy. The technology has been shown in preliminary studies to be associated with a high negative predictive value (98%) for detection of bacteriuria and promising accuracy of Gram stain classification of the microorganisms (via metabolic profiling of the cells) (54). Pattern intends to further develop the technology to enable both identification and susceptibility testing, using techniques already developed for respiratory and blood culture specimens.

### Rapid molecular identification methods

Multiple assays are in development which build on the now well-established “syndromic” panels available for indications such as pneumonia, sepsis, and gastroenteritis. These assays may include detection of one or more bacterial identifications and/or antimicrobial resistance genes. Most technologies in development apply real-time, multiplexed PCRs, although several technologies that use alternative nucleic acid amplification techniques are also under development. The summary presented in Table 3 outlines the types of approaches being developed by industry but is not all-encompassing due to the rapid expansion of interest in this market.

As has been the case for other syndromic panels, there is some challenge in defining the best use case for these technologies, as evidenced by the detection targets across the systems. Most of the assays in Table 3 include bacterial targets that are commonly isolated from both cases of uUTI and cUTI, including members of the Enterobacterales, *Pseudomonas aeruginosa*, and *Enterococcus* spp. Others also include pathogens typically seen only in uncomplicated infections (e.g., *Staphylococcus saprophyticus*), and some include detection of microorganisms present in specific populations, such as Group B *Streptococcus* in pregnant persons, or *Candida* spp. in diabetic patients. In all cases, the target lists are not finalized, as these platforms have not yet entered clinical trials for US FDA clearance.

It is important to highlight the variability in limit of detection (LOD) for each of the assays in Table 3, which range from 100 to 10,000 CFU/mL equivalent. The assay’s LOD (and availability of relative quantitation) is an important feature when determining the best use case for these assays, across patient populations. For instance, detection of low-level microorganisms (below 10^4^ CFU/mL) may be appropriate for patients who are clearly symptomatic with negative standard cultures, but inappropriate for patients as a first line for uncomplicated UTI, due to the risk of clinically false-positive results.

Antimicrobial resistance gene targets are included in most assays. These range from standard detection of *mecA*, *vanA,* and *vanB* to detection of multiple Gram-negative beta-lactamases, such as *bla*_CTX-M_ and the carbapenemase genes *bla*_KPC_, *bla*_NDM_, *bla*_VIM_, *bla*_IMP_, and *bla*_OXA-48_. Detection of these antimicrobial resistance genes, which are highly applicable to bloodstream infections (72), are unstudied in urine specimens. *S. aureus* only rarely causes true uncomplicated UTI, and treatment is generally doxycycline or trimethoprim, both of which maintain high rates of susceptibility among methicillin-resistant *S. aureus* (MRSA). However, *S. aureus* detection in the urine may indicate a disseminated infection or complicated UTI, in which case, knowledge of *mecA* is of value to determine vancomycin use (73). In contrast, UTI caused by vancomycin-resistant *Enterococcus* (VRE) are often treated with nitrofurantoin or doxycycline, both of which are high level of activity against VRE (74), or aminopenicillins, which retain clinical activity for uncomplicated UTI, despite MICs above the susceptible breakpoint (75). In this case, knowledge of *vanA*/*vanB* status may provide little clinical benefit. For Gram-negative infections, knowledge of CTX-M may not be value-added for uncomplicated UTI, but provides direction to treatment of cUTI, and pyelonephritis (76). As such, detection of additional targets for antimicrobial resistance outside those used in blood culture molecular tests would be of value for assays targeting urine specimens, as is planned by some tests in development (e.g., Unyvero UTI Panel, Curetis GmbH, Holzgerlingen, Germany; PathoKey UTI ID&AMR, Vela Diagnostics, Fairfield, New Jersey; and the Vivalytic UTI Test, Randox, Crumlin, UK). These assays include resistance markers for antimicrobial agents commonly used for treatment of UTI, such as sulfonamides (*sul1*) and/or fluoroquinolones (*qnrB*, *qnrS*). The prediction of resistance to these agents by evaluating a limited number of resistance gene targets is unclear, although it is likely to vary regionally, as the prevalence of these markers may vary by geography. For example, trimethoprim-sulfamethoxazole resistance may be due to *dfrA* and fluoroquinolone resistance is most commonly due to mutation to DNA gyrase, rather than the *qnr* plasmid-mediated mechanisms targeted by these assays (77).

Several assays in development have been designed with an eye to point-of-care testing. The Lodestar Dx UTI test (Llusern, Cardiff, UK) evaluates for six bacterial targets in urine using loop-mediated amplification, with a projected limit of detection of 10^4^ CFU/mL in 0.5 hours. The CLoNET *E. coli* assay (ID Genomics, Seattle, WA) detects *E. coli,* the most common cause of uUTI. The Vivalytic UTI RUO Test by Randox (Crumlin, UK) is also designed with point-of-care testing in mind; this panel evaluates for 20 bacterial targets and eight resistance genes in under 3 hours.

There are virtually no peer-reviewed data on the performance of these molecular assays. However, molecular detection of microorganisms in urine specimens has been an area of active research for several years, which provides some generalities with regard to nucleic acid amplification tests for UTI. A meta-analysis of studies evaluating the performance of PCR assays on urinary specimens for diagnosis of UTI showed an estimated sensitivity of 80.8% (73.4%–86.6%) and specificity of 83.7% (52.7%–95.9%) for this approach, as compared to standard cultures. It must be noted that these data are not based on the commercial systems in development. Nonetheless, the meta-analysis reported a false-positive rate of ~16%, which highlights the concern that molecular testing applied to urine specimens may yield unacceptably low specificity (55). This is of particular concern for patients with high incidence of ASB, such those in post-acute and long-term care facilities. Importantly, in this population, up to 50% of antimicrobial prescriptions are inappropriate due to treatment of asymptomatic bacteriuria; use of a nucleic acid amplification test (NAAT) for diagnosis of UTI has the risk of driving this rate of inappropriate antimicrobial use even higher (56). This concern has led to several position papers on the risks associated with use of NAAT for testing of patients in long-term care (57, 58), and specific guidance from the Washington State Department of Health and Washington State Society for Post-Acute and Long-Term Care Medicine to order traditional cultures, not PCR, for this population (https://doh.wa.gov/sites/default/files/2023-11/420-548-UrinePCRGuidanceWAPALTC-WADOH_0.pdf) Raising the limit of detection for reporting to levels clinically actionable (e.g., only report at crossing thresholds that correlate with culture results of ≥10^4^ or ≥10^5^ CFU/mL) or reporting relative quantification may help improve specificity of these assays. No data are published in this regard for urine specimens, but similar approaches for lower respiratory tract specimens show imperfect correlation with cultures and only moderate improvements in positive predictive values with semi-quantitative approaches (78).

Conversely, PCR-based technologies may provide some value to patients with complex and recurrent true UTI, as they are better able to detect fastidious microorganisms, such as *Actinotignum schaalii*, *Mycoplasma hominis*, or *Ureaplasma* spp. (79), if these are included among the targets of the panel. These targets may be important for some populations, particularly post-menopausal patients with recurrent UTI (80). However, differentiation of these as pathogens vs normal urinary microbiota complicates use of PCR in these instances (36). One study showed PCR was better able to detect low-level infections caused by typical organisms, including *E. coli* (81).

To date, no clear studies have defined the best population for which these technologies might be applied. This is largely due to the variability in assay design, which makes comparison across studies complex. Unsurprisingly, molecular assays with a larger breadth of targets are associated with increased detection of pathogens and species diversity, although no studies evaluated impact of these on patient management (59).

### Next-generation sequencing methods

Multiple companies are developing next-generation sequencing approaches for the assessment of microorganisms in urine and other specimens. The majority of these are offered only as reference laboratory models, as described further below. However, Illumina (San Diego, CA) has a urine-specific research use-only test available, the Urinary Pathogen ID/AMR (UPIP) enrichment kit. This test applies target-capture enriched next-generation sequencing to detect 121 bacterial species, 3,728 antimicrobial resistance genes, 14 fungi, 4 parasites, and 35 viruses within 24 hours (60).

Many studies exist in the literature that demonstrate application of NGS to urine specimens to aid with diagnosis of UTI. The substantial variability across the methodologies, subjects, syndromes, and study design across these studies makes drawing conclusions challenging. A recent meta-analyses was conducted that evaluated studies with similar design for the evaluation of NGS technologies in the diagnosis of UTI (59). This analysis included seven studies with 274 urine specimens from 262 patients. As expected, 78.1% of the 82 culture-negative specimens were positive by NGS. The species diversity was significantly higher in specimens when tested by NGS than by routine culture, with 170 bacterial taxa detected by NGS, only 38 of which were cultivable by standard culture (59). Only two studies in this analysis included AST predictions, which were concordant for 84.6% of 52 specimens analyzed (59).

As is the case for the NAAT assays described above, very limited data are available regarding clinical outcomes for the UPIP technology. However, several studies are available in the literature that assess NGS methods for diagnosis of UTI. It must be noted that most are associated with high risk of bias, as assessed by Cochrane risk of bias tool and the Newcastle Ottawa Scale (59). One illustrative study compared outcomes for patients with symptoms of acute cystitis whose treatment was guided by NGS versus standard-of-care urine culture. All 44 patients in this study had positive NGS results but only 13 had positive cultures. UTI symptom assessment scores showed greater symptomatic improvements in the 22 patients treated based on NGS *versus* the 22 treated by culture results (average net improvement, 8.5 vs 3.7, *P* < 0.001) (61). However, this study also enrolled 22 asymptomatic control patients, 5 of whom had positive urine culture results and 21 had positive NGS results, at an average of 10^5^ CFU/mL equivalents per specimen (61). These data show the importance of diagnostic stewardship for the use of NGS for the diagnosis of UTI; general consensus is that NGS testing provides no additional benefit to culture for patients with simple cystitis, but may be of use for patients with more complicated or recurrent infections, including interstitial cystitis, overactive bladder syndrome, bladder pain syndrome (62), where data are emerging that microorganisms non-cultivable by standard urine cultures are at play (63).

### Rapid phenotypic susceptibility tests

The development of more rapid, phenotypic AST methods has progressed substantially over the last 5 years, with tests available that perform MIC testing directly on blood culture broth or colony growth that yield results within 4–8 hours (82). These technologies are now being investigated for urine specimens, as shown in Table 4. Methods range from single-cell imaging, light scattering, and detection of enzymatic activity. Importantly, many of these technologies do not include an organism identification method, but rather only antimicrobial susceptibility results. The need for organism identification, particularly for uncomplicated cystitis, is debated by experts (83). However, the clinical ramifications of performing AST with no organism identification have not been evaluated, and current College of American Pathologists accreditation standards require AST to be linked to a specific organism on the final laboratory report.

The Astrego (Uppsala, Sweden) AST technology relies on nanofluidics, whereby bacterial growth is measured as a function of the length extension of individual cells as they divide within nanochannel microbial traps. The system does not provide bacterial identification but detects presence of bacteriuria as part of cell imaging. Early publications on this method show the system evaluates the unique morphological changes that bacteria undergo, as they proceed through the cell cycle, which are translated into growth rates. The unique microfluidic chip design serves as a filter for eukaryotic cells, as they are too large to enter the microfluidic cell channels. Initial evaluation showed good agreement with reference methods for ciprofloxacin susceptibility, within 30 minutes (64). A subsequent study which evaluated 278 women with suspected cystitis showed sensitivity and specificity for bacteriuria were 84% and 99%, respectively. Overall categorical agreement ranged from 85% for ciprofloxacin to 96% for trimethoprim in this study (65). Astrego has partnered with Sysmex (Kobe, Japan), the manufacturer of widely used urinalysis analyzers, for distribution of their product (66). Astrego was awarded the Longitude prize for antimicrobial resistance research in 2024 (https://amr.longitudeprize.org/teams/sysmex-astrego-ab/), and clinical studies are ongoing for this device.

The Alfred 60/AST (Alifax, Polverara, Italy) system is a well-vetted methodology based on laser light scattering which evaluates bacterial growth culture kinetics in the presence of antimicrobials, as compared to a growth control. The Alfred AST system is used widely throughout Europe for urine and other specimen types (49) but has not yet achieved FDA clearance for US use.

The ASTsense system (Module Innovations, Pune, India) is based on a technology called size-dependent signal amplification by nanoparticles. The technology involves use of gold nanoparticles conjugated to both antibodies specific to bacteria found in UTI and glucose oxidase. The nanoparticles are incubated with the patient’s urine for 30–90 minutes and then washed to remove unbound nanoparticles. Antimicrobial susceptibility is determined via colorimetric change through addition of glucose and further gold nanoparticles, the intensity of the color change is correlated to increasing antimicrobial resistance (67). The authors were unable to find any publications or data in the public domain regarding the performance of this technology. Module Innovations was funded by Carb-X to further develop their cartridge-based platform.

JIDDU system, in development by Astek Diagnostics (Baltimore, MD), isolates bacteria from urine using microfluidics and measures their metabolic activity using fluorescent dye detectors, which is used as an endpoint for AST determination. Little data are available on this test’s performance, but the company is targeting testing of antimicrobials commonly used for Gram-negative UTI, including trimethoprim/sulfamethoxazole, ceftriaxone, levofloxacin, nitrofurantoin, and cephalexin (https://astekdx.com/solution).

The iFAST system (iFast Diagnostics, Southampton, UK) measures changes in the electrical and morphological properties of bacteria using impedance flow cytometry (68). The assay’s readout is a measure of bacterial cell health (opacity) and size, which are then translated into antimicrobial susceptibility by comparing to a growth control. While iFAST first plans to launch their test on blood culture specimens, urine targets are an early milestone in their product roadmap. Early data of 58 *E. coli* and *K. pneumoniae* isolates against a critical concentration (i.e., at the resistance breakpoint) of amoxicillin-clavulanic acid, amoxicillin, ceftazidime, cephalexin, ciprofloxacin, gentamicin, nitrofurantoin, and trimethoprim showed 95.2% concordance with reference broth microdilution (BMD). All errors were for isolates with MICs within 1 log_2_ dilution of the breakpoint concentration. Results were available in 5 hours, in this pilot study (69).

U-DETECT (BioAmp Diagnostics, San Carlos, CA) technology is a dual-enzyme amplification method, which is specific to extended-spectrum beta-lactamases (ESBLs) present in a patient’s urine specimen. In this assay, the ESBL is connected to a caged enzyme amplifier through a beta-lactamase targeting probe. Upon ESBL hydrolysis, the caged enzyme amplifier is released, leading to a colorimetric signal output. In this way, the signal generated through ESBL activity is amplified up to 40,000-fold (70). While this technology is relatively limited in scope for AST, it does provide a rapid, inexpensive screen for ESBL activity, enabling rapid treatment decisions.

## POTENTIAL CAVEATS OF NEW DIAGNOSTICS: *IN VITRO* DIAGNOSTIC (IVD) AND NON-IVD PATHWAYS

As the preceding sections show, diverse new assays stand to expand current diagnostic options for UTIs. A variety of these products may continue toward larger clinical trials and formal regulatory approval, which in the USA entails approval/clearance by the FDA as an *in vitro* diagnostic. Although the underlying technologies are diverse, several common principles would apply to implementation of these tests in real-world medical practice, including potential challenges. Fundamentally, the goal of any new diagnostic is to improve upon existing standards of care, especially in terms of increased sensitivity, specificity, or speed. Although there is a natural desire to apply emerging technologies to longstanding clinical challenges, molecular diagnostics are not intrinsically superior to other methodologies. The specific context of testing is critical to gauging added value. Ambiguity around detection of particular species at a CFU/mL level in a urine culture equally applies to that organism’s molecular detection. More so, the need for cross-calibration between culture and molecular techniques entails a further level of complexity, especially with the ability of NAATs to detect nonviable or quiescent bacteria.

In addition to clinical and analytic factors, new diagnostics must always be viewed through the broader lens of healthcare systems. The potential clinical value of new technologies are always weighed against their financial implications for the patient, provider, payer, and laboratory. This dynamic is already at play for syndromic molecular testing for respiratory and gastrointestinal pathogens, which are generally only reimbursed for a narrow set of indications (https://www.cms.gov/medicare-coverage-database/view/article.aspx?articleid=58710&ver=39&). Relative to traditional culture, multitarget molecular assays might entail more favorable reimbursement to the performing laboratory, but their appropriateness for different patient populations is a function of cost-benefit analyses across these stakeholders.

These considerations would certainly apply to any novel UTI diagnostics that achieve IVD status. At the same time, we must acknowledge the parallel growth in laboratory-developed tests (LDTs) offered through specialized reference laboratories, already in clinical use, for which such caveats may be further magnified. In the USA, for instance, Clinical and Laboratory Improvement Amendment-accredited laboratories have historically been given leeway to design, validate, and market in-lab-developed tests without direct review by the FDA (and subsequent IVD status) under a model of enforcement discretion (84). Accordingly, various commercial reference laboratories have developed and promoted LDTs for the evaluation of microbes in urine (see Table 5). These include assays that ostensibly identify and/or quantify numerous uropathogens through nucleic acid testing. In addition, at least two laboratories (Table 5) provide a “pooled” antimicrobial susceptibility assessment, whereby a mixture of microorganisms is subjected to proprietary AST in one test. A common anecdotal phenomenon, in some cases, is the direct marketing of these assays to providers (including the potential for direct third-party billing), bypassing institutions’ clinical laboratory infrastructure. Admittedly, it is difficult to offer granular comments on all such LDTs, in some cases due to a paucity of peer-reviewed (or publicly available) data on their analytic characteristics, clinical performance, and underlying validation.

An example of a UTI LDT, which has been described in several publications, is the Guidance UTI test by Pathnostics; this assay also recently received approval by the Clinical Laboratory Evaluation Program of the New York State Department of Health (an additional state-specific LDT requirement in New York) (79, 85). The Guidance UTI test entails real-time PCR targeting bacterial genomic DNA from numerous organism classes (30 total), reported in cells/mL-units that emulate semi-quantitative culture results. These organisms cover both classically recognized uropathogens, as well as various members of the urobiome discussed above, which have been described as both occasional emerging uropathogens, as well physiologic commensals, potentially on a patient-by-patient basis. The latter include *A. schaalii*, *A. omnicolens*, *G. vaginalis*, *C. urealyticum*, coagulase-negative staphylococci, viridans streptococci, and various *Candida* species. The Guidance UTI assay likewise includes the PCR detection of various genetic determinants of antimicrobial resistance, in a source-independent manner, along with the aforementioned process of pooled AST. In general, the clinician-reportable results from such LDTs can include an analytic summary of the data generated by that test—in however manner it was internally validated—but it remains at the discretion of the provider to interpret their significance and decide what to treat, which may be challenging for organisms where clinical guidelines do not exist. Some reference laboratories that offer such tests attempt to improve interpretation through test reporting, which may include suggestions on antimicrobials that may cover the detected organisms, although these recommendations are disclaimed as not patient specific. Data used as part of marketing for the Guidance UTI includes evaluation of only 69 patients (https://pathnostics.com/tests/guidance-uti/), and include claims for reduced rates of outpatient emergency room visit, hospital admissions, and a projected reduction in cost per patient. These findings have not been corroborated by independent studies.

Finally, at least in the United States, it also critical to comment on the general landscape of LDTs, which are in a time of significant potential change. Just this year, the FDA announced plans to begin phasing in more direct oversight of LDTs performed by individual clinical laboratories (86). While this dynamic is still actively evolving, it is expected that many assays—in particular diagnostics that are not internal to a particular healthcare system—will soon be subject to greater review. How this shift impacts the future availability and reporting of molecular LDTs for uropathogens remains an unresolved question.

## CONCLUDING THOUGHTS AND FUTURE DIRECTIONS

While urine culture has long served as a cornerstone for diagnosing UTI, its limitations have paved the way for the development of new and advanced laboratory technologies. Nevertheless, to incorporate these tools into clinical practice, they must be harmonized with emerging knowledge on the complexity of urinary microbiology. In some patients, for example, UTIs can occur at low microbial levels, even below typical diagnostic thresholds, while others may carry high microbial burdens without symptomatology. Similarly, we must reconcile the traditional interpretation of multi-species detection (as contamination) with the fact that polymicrobial infections also represent a clinical reality. These include polymicrobial scenarios in which the urobiome is associated with pathology that does not conform to traditional paradigms of infection. In general, there is a tremendous need for outcomes research that defines the proper utilization of new assays amid the increasing complexity of clinical urology. Ideally, to leverage advanced diagnostics in real-world practice, the field must define the indication for such methods in the context of existing framework and definitions (UTI versus ASB, upper vs lower, uUTI versus cUTI *et cetera*). Only in this way can powerful analytic technologies be utilized for maximum diagnostic value.
